# Disability pension among gynaecological cancer survivors with or without radiation-induced survivorship syndromes

**DOI:** 10.1007/s11764-021-01077-9

**Published:** 2021-08-19

**Authors:** Adnan Noor Baloch, Mats Hagberg, Sara Thomée, Gunnar Steineck, Helena Sandén

**Affiliations:** 1 Occupational and Environmental Medicine, School of Public Health and Community Medicine, Institute of Medicine at University of Gothenburg, P.O. Box 463, 405 30 Gothenburg, Sweden; 2 Department of Psychology, University of Gothenburg, Box 500, 405 30 Gothenburg, Sweden; 3 Division of Clinical Cancer Epidemiology, Department of Oncology, Institute of Clinical Sciences at University of Gothenburg, 405 30 Gothenburg, Sweden

**Keywords:** Cancer survivor, Radiotherapy/adverse effects, Disability pension, Radiation-induced syndromes, Return to work, Faecal incontinence

## Abstract

**Purpose:**

Gynaecological cancer patients treated with external radiation therapy to the pelvis may face long-lasting and long-term gastrointestinal syndromes. The aim of this study was to assess the association between such radiation-induced survivorship syndromes and disability pension among gynaecological cancer survivors treated with pelvic radiation therapy.

**Methods:**

This prospective register study included gynaecological cancer survivors (n=247) treated during 1991–2003, alive at the time of the study, and <65 years of age. In 2006, they completed a postal questionnaire measuring patient-reported outcomes. The self-reported data were linked to the national register on disability pensions. Relative risks and risk differences with 95% confidence intervals (CIs) of being granted a disability pension were estimated using log-binomial regression.

**Results:**

Gynaecological cancer survivors with gastrointestinal syndromes had a higher risk of disability pension than survivors without such syndromes. Survivors with blood discharge syndrome had a 2.0 (95% CI 1.3–3.2) times higher risk of disability pension than survivors without blood discharge syndrome. The relative risk among survivors with urgency syndrome was 1.9 (1.3–2.9) and for leakage syndrome, 2.1 (1.4–3.1). Adjusting for age did not affect our interpretation of the results.

**Conclusions:**

Gynaecological cancer survivors with a specific radiation-induced survivorship syndrome have a higher risk of disability pension than survivors without that specific syndrome.

**Implications for Cancer Survivors:**

The findings highlight the need for more awareness and knowledge regarding the potential role of radiation-induced survivorship syndromes for continuing work among gynaecological cancer survivors. Work-life-related parameters should be considered during radiotherapy and rehabilitation after treatment.

**Supplementary Information:**

The online version contains supplementary material available at 10.1007/s11764-021-01077-9.

## Introduction

For a woman diagnosed with gynaecological cancer, a few weeks of cancer treatment may determine how she fares for the rest of her life. The treatments may give rise to lifelong, treatment-induced conditions, all adverse in some way [1–5]. Radiation therapy to cure cancer in pelvis also affects the gastrointestinal health of cancer patients [1, 6]. Consequently, cancer survivors suffer from gastrointestinal symptoms with an adverse effect on their quality of life [4, 6–8] and workability [7, 9]. Utilizing better technology to deliver doses of radiation precisely and effectively can prevent these gastrointestinal symptoms. This may increase costs for the care provider in the short term but may lead to lower costs for the individual and society in the long term. In addition, the cessation of suffering for a cancer survivor will be the sought after win. To move forward, we need to learn more about economically relevant outcomes concerning the downside of cancer treatment. Disability pension is one such cost.

The Swedish disability pension is equivalent to the Social Security Disability Insurance (SSDI) in the USA or to the Employment and Support Allowance (ESA) in the UK. It is granted to individuals with income from work or unemployment benefits, and with failing health resulting in reduced work capacity of at least 25% [10]. At the same time, a person placed on disability pension may suffer from new health risks. The loss of social networking with peers or a regular schedule can lead to unhealthy living habits and even depression [11]. A cancer survivor placed on disability pension therefore faces two health hazards, the treatment-induced condition and the effects associated with being on disability pension. We know that women who have been cured of gynaecological cancer are more likely than other women to receive a disability pension [12, 13]. However, we do not know which treatment-induced conditions mediate this effect. If we can identify these mediators, we have a basis to implement cost-effective preventive measures in connection with cancer treatment.

Each Swedish resident has a unique personal identity number (PIN). This number allows us to link questionnaire-derived information and clinical information to administrative records for any individual on disability pension. This has made it possible for us to determine the extent to which the intensity of five different radiation-induced syndromes, each decreasing intestinal health, affects the likelihood of being granted a disability pension.

## Methods

### Study design

This study had a prospective cohort design. At baseline in 2006, the initial patient-reported outcomes were collected and analyzed to identify the predictors (radiation-induced survivorship syndromes). The outcome, disability pension, was investigated for the year 2008 (2-year follow-up). The baseline data and the analyses identifying the specific cancer-survivor syndromes are described in detail in a previous study [14].

### Participants

Dunberger et al. describe the data collection in detail [15, 16]. In short, a clinical patient cohort of 1,800 women treated during 1991–2003 with external pelvic radiotherapy for a gynaecological cancer malignancy, either at Jubileumskliniken, Sahlgrenska University Hospital in Gothenburg, or at Radiumhemmet, Karolinska University Hospital in Stockholm, Sweden, was identified through the medical records. The inclusion criteria of having been born in 1927 or later and being able to read and understand Swedish were met by 823 cancer survivors. A flowchart is provided in the supplementary information in Online Resource 1.

### Patient-reported outcomes of long-term pelvic radiation symptoms

Between January and October 2006, the 823 cancer survivors were sent an introductory letter explaining the study objectives and were individually contacted by telephone. Those giving informed consent during the telephone call (n=723) received a postal questionnaire. Three weeks after posting the questionnaire, a thank you and reminder card was sent to them. Where appropriate, a reminder telephone call was made. Altogether 650 cancer survivors returned the completed questionnaire, but seven were excluded because of missing information on syndromes, and 20 because of bowel stoma. All actions were taken by a neutral third party [17]; none of the previously involved health care professionals were involved or had access to the data.

The postal questionnaire consisted of 351 questions regarding demographics, intercurrent diseases and comorbidity, psychological issues, quality of life, and sexual function. In addition, respondents were asked about symptoms from the abdomen, the gastrointestinal tract, genitals, legs, pelvic bones, and urinary bladder. Furthermore, questions about the occurrence, intensity, and duration of the abovementioned symptoms were included. The development and validation process of the questionnaire is well documented [15].

### Predictor: radiation-induced survivorship syndromes

In a previous study [14], data on the cancer survivors and 344 matched controls were analyzed. Using a modified factor analysis approach, a total of 28 symptoms marking a decrease in intestinal health were found to be related to six factors. Steineck and co-workers interpreted and termed these factors *Radiation-induced survivorship syndromes* [14]. The factors are *urgency syndrome*, *leakage syndrome*, *constipation*, *excessive gas discharge syndrome*, *excessive mucus discharge syndrome*, and *blood discharge syndrome*. For all factors apart from *constipation*, there was a statistically significant difference between survivors and controls concerning factor score quantiles. We used these five syndromes and classified survivors as having a specific syndrome if their factor loading on that syndrome was above the 95th percentile of the controls, as outlined by Steineck et al. [14]. Cancer survivors could be classified as having more than one syndrome. To investigate whether the level of association differed between cancer survivors classified as having several syndromes and survivors classified as having one syndrome, or as having none, we summed up the number of syndromes reported for each survivor.

### Disability pension (outcome)

The personal identification number (PIN) of each of the cancer survivors was linked to the Longitudinal Integrated Database for Health Insurance and Labour Market Studies (LISA) held by Statistics Sweden. This database integrates existing data for individuals by retrieving information from official Swedish registries. It includes all individuals ≥16 years of age registered in the Total Population Register on 31st of December each year and covered by the social insurance system [10].

In the Swedish social security system, the requirements for receiving disability pension are being insured (legally living and/or legally working in Sweden), age 19–64 years, and full or partial reduction in capacity for work (by at least 25%) because of sickness or a disability, as evaluated and certified by an insurance physician. Students, unemployed individuals, and housewives are also entitled to disability pension. Disability pension can be combined with early old-age pension once the individual has turned 62 years old. The binary variable disability pension is either “granted” or “not granted” and can be designated as partial or full.

### Statistical analysis

We followed Statistical Analyses and Methods in Published Literature (SAMPL) [18] and Strengthening the Reporting of Observational Studies in Epidemiology (STROBE) [19] guidelines for this manuscript. For descriptive statistics, we used frequency counts and percentages. Simple log-binomial regressions were performed to assess the association between having a specific syndrome (yes/no) and disability pension. Besides the simple/un-adjusted models, multivariable log-binomial regressions adjusting for age (in years) were also performed. The sum of the number of syndromes was also used as a predictor in regression analysis. The assumptions of binary outcome data, sufficient events in syndrome categories, and independence of error were satisfied. The level of significance (alpha) was set at 5%. All hypothesis tests were two-sided and no adjustments were made for multiple statistical testing.

We reported both relative risks (RRs) and risk differences (RDs) along with their respective 95% confidence intervals (CIs). The association was considered significant when the corresponding 95% CIs were above 1 (for RR) and > 0 (for RD). We used SAS GENMOD procedure with binomial distribution and a log link for RR, and an identity link for RD. All statistical analyses were performed using SAS 9.4 (RRID:SCR_008567; SAS Institute, Cary, NC, USA).

### Patient and public involvement

The study-specific questionnaire was developed in close co-operation with gynaecological cancer survivors. They were interviewed in a semi-structured way, and they raised important questions and issues regarding radiation therapy, symptoms, quality of life, and social functioning. The study-specific questionnaire was face-validated and tested in a pilot study, where the majority of participants were cancer survivors. Cancer survivors perceived the study as valuable and said they were positively affected by their participation [20]. One of the researchers and co-authors of the present study is herself a gynaecological cancer survivor and a patient representative.

### Ethical declarations

This study was approved by the Regional Ethical Review Board in Gothenburg, Sweden (Reg. No. 671-17), and was conducted in accordance with the Declaration of Helsinki.

## Results

### Participants

A total of 247 gynaecological survivors from the initial cohort were eligible for the current study. The main reason for exclusion was age as only individuals aged 19–64 years are entitled to disability pension [10], and some occupational pensions are automatically paid at 65 years of age [21]. For details, see the flow chart (Online Resource 1). Some clinical and demographic characteristics for survivors are summarized in Table 1; more detailed descriptive statistics are provided in Online Resource 2.

**Table 1:** Baseline clinical and demographic data for all gynaecological cancer survivors and for gynaecological cancer survivors with and without syndromes

	All survivors	Gynaecological survivors with				Urgency syndrome		Leakage syndrome		Blood discharge syndrome	
		No syndrome	One syndrome	Two syndromes	Three syndromes	Yes	No	Yes	No	Yes	No
	n=247	n=131 (53 %)^a^	n=50 (20%)^a^	n=49 (20%)^a^	n=17 (7 %)^a^	n=91 (37 %)^a^	n=156 (63 %) ^a^	n=77 (31 %) ^a^	n=170 (69 %)^a^	n=31 (13 %)^a^	n=216 (87 %)^a^
	No. (%)^﻿b^	No. (%)^﻿b^	No. (%)^﻿b^	No. (%)^﻿b^	No. (%)^﻿b^	No. (%)^﻿b^	No. (%)^﻿b^	No. (%)^﻿b^	No. (%)^﻿b^	No. (%)^﻿b^	No. (%)^﻿b^
Age											
16–29 years	2 (1 %)	0	2 (4 %)	0	0	2 (2 %)	0	0	2 (1 %)	0	2 (1 %)
30–49 years	66 (27 %)	36 (27 %)	13 (26 %)	14 (29 %)	3 (18 %)	24 (26 %)	42 (27 %)	19 (25 %)	47 (28 %)	7 (23 %)	59 (27 %)
50–64 years	179 (72 %)	95 (73 %)	35 (70 %)	35 (71 %)	14 (82 %)	65 (71 %)	114 (73 %)	58 (75 %)	121 (71 %)	24 (77 %)	155 (72 %)
Self-reported employment status											
Employed	169 (69 %)	100 (77 %)	33 (69 %)	28 (57 %)	8 (47 %)	54 (59 %)	115 (75 %)	43 (57 %)	126 (75 %)	16 (53 %)	153 (72 %)
Disability pension	35 (14 %)	10 (8 %)	5 (10 %)	14 (29 %)	6 (35 %)	22 (24 %)	13 (9 %)	20 (26 %)	15 (9 %)	9 (30 %)	26 (12 %)
Unemployed	12 (5%)	7 (5 %)	2 (4 %)	2 (4 %)	1 (6 %)	3 (3 %)	9 (6 %)	4 (5 %)	8 (5 %)	2 (7 %)	10 (5 %)
Housewife, other	10 (4 %)	5 (4 %)	2 (4 %)	2 (4 %)	1 (6 %)	3 (3 %)	7 (5 %)	5 (7 %)	5 (3 %)	1 (3 %)	9 (4 %)
Sickness absence	9 (4 %)	2 (2 %)	5 (10 %)	2 (4 %)	0	7 (8 %)	2 (1 %)	1 (1 %)	8 (5 %)	1 (3 %)	8 (4 % )
Student	5 (2 %)	3 (2 %)	0	1 (2 %)	1 (6 %)	2 (2 %)	3 (2 %)	2 (3 %)	3 (2 %)	1 (3 %)	4 (2 %)
Retired	4 (2 %)	3 (2 %)	1 (2 %)	0	0	0	4 (3 %)	1 (1 %)	3 (2 %)	0	4 (2 %)
Not stated	3	1	2	0	0		3	1	2	1	2
Diagnosis											
Endometrial cancer	104 (42 %)	57 (44 %)	24 (48 %)	19 (39 %)	4 (24 %)	38 (42 %)	66 (42 %)	27 (35 %)	77 (45 %)	9 (29 %)	95 (44 %)
Cervical cancer	93 (38 %)	52 (40 %)	17 (34 %)	18 (37 %)	6 (35 %)	33 (36 %)	60 (38 %)	27 (35 %)	66 (39 %)	11 (35 %)	82 (38 %)
Ovarian cancer	23 (9 %)	10 (8 %)	3 (6 %)	8 (16 %)	2 (12 %)	10 (11 %)	13 (8 %)	11 (14 %)	12 (7 %)	4 (13 %)	19 (9 %)
Vaginal cancer	11 (4 %)	4 (3 %)	3 (6 %)	1 (2 %)	3 (18 %)	4 (4 %)	7 (4 %)	5 (6 %)	6 (4 %)	5 (16 %)	6 (3 %)
Sarcoma uteri	9 (4 %)	5 (4 %)	2 (4 %)	2 (4 %)	0	3 (3 %)	6 (4 %)	3 (4 %)	6 (4 %)	0	9 (4 %)
Fallopian tube cancer	5 (2 %)	2 (2 %)	1 (2 %)	1 (2 %)	1 (6 %)	2 (2 %)	3 (2 %)	3 (4 %)	2 (1 %)	1 (3 %)	4 (2 %)
Vulvar cancer	2 (1 %)	1 (1 %)	0	0	1 (6 %)	1 (1 %)	1 (1 %)	1 (1 %)	1 (1 %)	1 (3 %)	1 (1 %)
Treatment modality											
Surgery + EBRT^c^ + BT^d^	110 (45 %)	66 (51 %)	23 (46 %)	18 (37 %)	3 (18 %)	35 (38 %)	75 (48 %)	24 (31 %)	86 (51 %)	9 (29 %)	101 (47 %)
Surgery + EBRT^c^ + BT^d^ + Chemo^e^	56 (23 %)	30 (23 %)	17 (34 %)	5 (10 %)	4 (24 %)	18 (20 %)	38 (25 %)	15 (19 %)	41 (24 %)	6 (19 %)	50 (23 %)
Surgery + EBRT^c^ + Chemo^e^	28 (11 %)	11 (8 %)	4 (8 %)	9 (18 %)	4 (24 %)	14 (15 %)	14 (9 %)	15 (19 %)	13 (8 %)	5 (16 %)	23 (11 %)
Surgery + EBRT^c^	18 (7 %)	7 (5 %)	4 (8 %)	5 (10 %)	2 (12 %)	8 (9 %)	10 (6 %)	7 (9 %)	11 (7 %)	5 (16 %)	13 (6 %)
EBRT^c^ + BT^d^ + Chemo^e^	17 (7 %)	11 (8 %)	1 (2 %)	5 (10 %)	0	5 (5 %)	12 (8 %)	4 (5 %)	13 (8 %)	2 (6 %)	15 (7 %)
EBRT^c^ + BT^d^	11 (4 %)	5 (4 %)	1 (2 %)	4 (8 %)	1 (6 %)	5 (5 %)	6 (4 %)	6 (8 %)	5 (3 %)	1 (3 %)	10 (5 %)
EBRT^c^ + Chemo^e^	5 (2 %)	0	0	3 (6 %)	2 (12 %)	5 (5 %)	0	5 (6 %)	0	2 (6 %)	3 (1 %)
EBRT^c^	1 (<1 %)	0	0	0	1 (6 %)	1 (1 %)	0	1 (1 %)	0	1 (3 %)	0
Not stated	1	1					1				1
Parity											
Never given birth	80 (32 %)	47 (36 %)	14 (28 %)	15 (31 %)	4 (24 %)	27 (30 %)	53 (34 %)	21 (27 %)	59 (35 %)	8 (26 %)	72 (33 %)
1–3 children	151 (61 %)	77 (59 %)	32 (64 %)	31 (63 %)	11 (65 %)	56 (62 %)	95 (61 %)	52 (68 %)	99 (58 %)	19 (61 %)	132 (61 %)
> 3 children	16 (6 %)	7 (5 %)	4 (8 %)	3 (6 %)	2 (12 %)	8 (9 %)	8 (5 %)	4 (5 %)	12 (7 %)	4 (13 %)	12 (6 %)

^a^Number (percentage) within each category of syndrome

^b^Number and percentage of survivors in each category

EBRT^c^ denotes external beam radiation therapy

BT^d^ denotes brachytherapy

Chemo^e^ denotes chemotherapy

### Radiation-induced survivorship syndromes

Of the 247 survivors, 47 % (116) had at least one syndrome while 53% (131) did not have any syndromes. For each specific syndrome, the number of survivors categorized as having that specific syndrome differed; see Table 1.

### Outcome data

**Figure 1.**
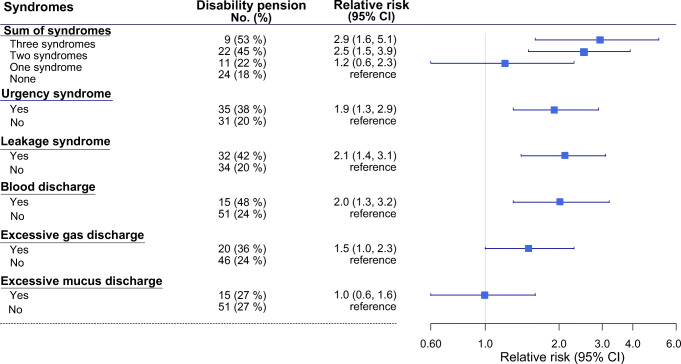
Number (percentage) and relative risk (RR) (95% confidence interval) of disability pension (data taken from the official register) at the 2-year follow-up. Relative risks (CIs) obtained from log-binomial regression analyses using *syndromes* as a predictor. Self-reported symptoms were used to classify survivors having a syndrome. A relative risk of > 1 indicates harm (No. = number)

At the 2-year follow-up, out of all gynaecological cancer survivors, 27% had been granted a disability pension. The percentage varied between the five syndromes and sum of syndromes, as shown in Figure 1 and Online Resource 3. Among survivors with urgency syndrome, 38% were granted disability pension, in contrast to only 20% of survivors without urgency syndrome, resulting in an RR of 1.9 (95% CI 1.3–3.0) and an RD of 19% (95% CI 7–30%); see Figure 1 and Table 2.

**Table 2:** Unadjusted relative risks (RRs) and risk differences (RDs) (95% confidence intervals (CIs)) for disability pension (from the national register on disability pension) among survivors with one or more syndromes

	Disability pension at 2-year follow-up			
	Relative risks^a^ (95 % CI)		Risk differences ^b^ (95% CI)	
Syndromes^c^	n = 247^d^	n = 243^e^	n = 247^d^	n = 243^e^
Sum of syndromes^﻿f^				
Three syndromes vs none	**2.9 (1.6 to 5.1)**	**2.9 (1.6 to 5.3)**	**35% (10% to 59%)**	**35% (10% to 60%)**
Two syndromes vs none	**2.5 (1.5 to 3.9)**	**2.5 (1.5 to 4.0)**	**27% (11% to 42%)**	**27% (12% to 42%)**
One syndrome vs None	1.2 (0.6 to 2.3)	1.2 (0.7 to 2.4)	4% (-10% to 17%)	4% (-9% to 18%)
Urgency vs no urgency syndrome	**1.9 (1.3 to 2.9)**	**2.0 (1.3 to 3.0)**	**19% (7% to 30%)**	**19% (7% to 31%)**
Leakage vs no leakage syndrome	**2.1 (1.4 to 3.1)**	**2.1 (1.4 to 3.1)**	**22% (9% to 34%)**	**22% (9% to 34%)**
Blood vs no blood discharge syndrome	**2.0 (1.3 to 3.2)**	**2.1 (1.3 to 3.2)**	**25% (6% to 43%)**	**25% (6% to 43%)**
Excessive gas vs no excessive gas discharge syndrome	1.5 (1.0 to 2.3)	1.5 (1.0 to 2.3)	12 % (−2% to 26%)	12 % (−2% to 27%)
Excessive mucus vs no excessive mucus discharge syndrome	1.0 (0.6 to 1.6)	1.0 (0.6 to 1.6)	0 % (−13% to 13%)	0 % (−13% to 13%)

^a,b^Unadjusted RR and RD (95% CI) obtained from log-binomial regression analyses using only a *Syndrome* as a predictor with ‘None/No’ level used as a reference

^c^Self-reported symptoms were used to build syndrome

^d^Survivors alive at follow-up in 2008

^e^Excluding survivors who died within the 2-years of follow-up (between 2008 and 2010)

^f^Survivors classified as having several syndromes or one or none. Bold numbers indicate a statistically significant association at 5% level of significance

Correspondingly, among survivors with blood discharge syndrome, an RR of 2.0 (95% CI 1.3–3.2) and an RD of 25% (95% CI 6–43%) was seen. Furthermore, among survivors with leakage syndrome, an RR of 2.1 (1.4–3.1) and an RD of 22% (95% CI 9–34%) were obtained. We observed a substantively important RD and RR of being granted disability pension for survivors with excessive gas discharge syndrome; however, the 95% CI for RD included the null value. The RR of being granted disability pension for survivors with excessive mucus discharge syndrome was 1.0 and the 95% CI included the null value. It was wide but extended to values that could be important; see Figure 1 and Table 2.

We also observed a higher RR of disability pension for survivors with one or more syndromes than for survivors without any syndromes. At the 2-year follow-up, survivors with two syndromes had an RR of 2.5 (95% CI 1.5–3.9) while survivors with three syndromes had an RR of 2.9 (95% CI 1.6–5.1) compared with survivors without any syndrome (see Table 2). Surprisingly, the increase in effect measures was monotonic. In addition, the Cochran-Armitage test, assuming sum of syndrome as an ordinal predictor and disability pension as a binary outcome, was significant (p<0.05) and supported the trend hypothesis.

In the multivariable regression analysis, age (in years) did not change our interpretation concerning the association between a syndrome and granting of a disability pension. These results are provided in Online Resource 4.

Gynaecological cancer survivors with a specific syndrome or specific syndromes were between 1.9 and 2.9 times more likely to be granted a disability pension than gynaecological cancer survivors without those syndromes. This was true even after adjusting for age in multivariable regression. The risk of being granted disability pension was highest among survivors classified as having three syndromes and next highest among those with blood discharge syndrome (Online Resource 3). The RDs with 95% CIs are shown in Online Resource 5. The sample size affects the width of the CI; because of the small sample size, the CIs were wide. Nevertheless, the point estimates are the best indicator of population-level values.

### Sensitivity analyses

We performed a sensitivity analysis by removing the survivors who died within 2 years of receiving disability pension and repeating the regression analyses. This step did not result in any remarkable changes in risk, RD or RR (see Table 2 and Online Resource 3).

## Discussion

This is the first prospective study to investigate the association between disability pension and radiation-induced survivorship syndromes in a national sample of gynaecological cancer survivors. The results of the study may be important because these radiation-induced survivorship syndromes are potentially modifiable. Their occurrence may be predicted from the knowledge of the dose of ionizing radiation delivered to each of a number of different parts of the intestine. By interlinking Swedish population-based registers, clinical data, and patient-reported outcomes, we found that gynaecological-cancer survivors with specific radiation-induced syndromes were more likely to receive disability pension than cancer survivors without such syndromes. Furthermore, the likelihood of being granted a disability pension increased with each increase in the number of radiation-induced syndromes.

Our first major finding was that gynaecological cancer survivors with one or more radiation-induced syndromes had about double the risk of being granted a disability pension compared with survivors who had no such syndrome. To the best of our knowledge, no other studies have investigated the relationship between specific radiation-induced syndromes and the risk of disability pension. There are previous studies that note a greater likelihood of being granted a disability pension in cancer survivors treated with radiation and/or chemotherapy; however, the magnitude of this risk is not known. A Swedish study [12] based on a large population-based cohort of gynaecological cancer survivors shows that cancer patients treated with radiotherapy and/or chemotherapy alone had a 1.7 times increased risk of being granted a disability pension compared with those treated with hysterectomy only. In a recent Norwegian study [13], long-term cervical cancer survivors were reported to have a two times higher prevalence of disability pension compared with females in the general population. In addition, a significantly higher proportion of cervical cancer survivors on disability pension reported treatment with pelvic radiation combined with chemotherapy compared with cervical cancer survivors in paid work [13]. Furthermore, in a large Danish population-based cohort study, patients with ovarian cancer had a 2.5 times higher risk of being granted an early retirement pension than population-based controls, and patients with cervical cancer had a 1.3 times greater risk than population-based controls [22]*.* These risks were adjusted for demographic, health-related characteristics, and sickness benefit in the year prior to early retirement. Moreover, a recent Italian study reported increased difficulty in returning to work among cancer survivors diagnosed with a malignant tumour and treated with radiation therapy [23]. Our data indicate that late adverse effects related to defaecation urgency, faecal leakage, or anal blood discharge may often be the most likely to lead to a disability pension.

There is some additional information in the literature that supports the notion that radiation-induced urgency syndrome and radiation-induced faecal leakage syndrome may decrease work ability in many cancer survivors, therefore increasing the risk of disability pension. We have, however, found no such supporting data in the literature concerning a relation between anal blood discharge and decrease in work ability. A large US household survey based on a representative sample of 5,400 adults found that 30% of individuals with gross faecal incontinence described themselves as being too unwell to work or go to school, as compared with 4.2% among individuals without any gastrointestinal symptoms [24]. A higher rate of work absenteeism is reported among individuals with gross faecal incontinence than in individuals without experiencing this symptom [24]. Cancer survivors receiving radiation therapy are more likely to report limitations in physical capabilities and job performance than cancer survivors receiving other kinds of treatment [25]. Fatigue, lack of energy, and keeping up with others were among the concerns of these survivors [25]. Dunberg et al. revealed that faecal incontinence among gynaecological cancer survivors was self-reported to negatively affect social functioning, ability to work, and quality of life [16]. Studies employing randomized controlled trials of radiotherapy for endometrial cancer have reported an increase in bowel symptoms among patients treated with pelvic radiation therapy [5, 26]. These symptoms have been reported to lead to increased limitations in daily activities and a higher need to remain close to a toilet, resulting in a lower level of social activity compared with patients not treated with radiation therapy [5, 26].

Our second major finding was that the greater the number of radiation-induced syndromes, the greater the risk of needing a disability pension. Females with both urinary and anal incontinence have reported a worse quality of life and greater impairment of physical functioning than females with only urinary incontinence [27, 28]. In a randomized treatment trial among prostate cancer patients, an increased symptom burden was associated with a lower quality of life and sense of well-being [29]. An increased number of long-term adverse effects of cancer treatments (surgery, radiotherapy, chemotherapy, endocrine) were also reported to be associated with decreased work ability and/or being non-employed among cancer survivors who had mixed diagnoses [30]. Therefore, it is likely that the higher the number of treatment-related syndromes, the higher is the impact on the employment and the work ability of cancer survivors. Thereby, an increasing number of radiation-induced syndromes are further limiting participation in working life.

### Strengths

One of the many strengths of our study is the large population-based patient cohort from two large hospitals in Sweden which together serve a catchment population of 3.5 million people. In Sweden’s government-funded universal health care system, all women diagnosed with a gynaecological cancer are referred to a specific radiotherapy hospital based on their place of residence.

Also, rather than simply using 28 long-term intestinal symptoms, we used modified factor analysis to relate these to six factors termed *radiation-induced survivorship syndromes* [14].

Furthermore, by consulting official records of disability pensions we assessed associations of these syndromes with disability pension. This reduced the effect of common sources of bias in prospective research, such as attrition.

### Limitations

When it comes to confounding, it should be mentioned that a potential weakness is that individuals who have these syndromes may have had their radiation therapy delivered in a different, and perhaps inferior, way to patients who do not have these syndromes. Against this backdrop, we found that there was no increased risk of disability pension with increased mucus secretion or flatulence. We did not assess the different combinations of syndromes due to lack of statistical power. There is a possibility that the risk of disability pension among cancer survivors in this study has been underestimated because of the death of survivors before they could answer the questionnaire, assuming that the remaining survivors have had better health than the deceased ones and also than those who had already quit working before our study was conducted. In our sensitivity analysis, we did not discover any notable changes in results.

### Drop-out rate

The drop-out rate in this study is considered relatively low and there is no indication of any selective drop-out that may have affected the results of the study.

### Misclassification

We consider that misclassification of the radiation-induced syndromes and disability pension is fairly low; however, any misclassification will result in dilution of the measure of co-variation we studied. If we had managed to minimize the misclassification, our results would probably have been even more precise.

We were unable to distinguish between survivors receiving part-time disability pension benefits along with part-time work and those receiving full-time disability pension benefits because of leaving the job market. Even though the patient sample was population-based, it only contained working-aged individuals, making the findings generalizable only to this group. In addition, findings from this study may be most generalizable to countries with social insurance systems comparable to that of Sweden, and less to other countries. Disability pension is a robust outcome of work disability as this state compensation requires an insurance physician to evaluate activity impairment based on the patient records. However, one can expect functional limitations and an impaired work ability among gynaecological cancer survivors with one or more of the studied syndromes even in other settings. We would also like to point out that the official register used was not specifically developed for research but was formed to be used for administrative purposes in health care and social welfare.

### Unanswered questions

Studies of aspects of occupational life many years after cancer treatment, quantitative studies, or studies using mixed methods to combine survivors’ perspectives with quantitative data on work and/or work ability and radiation-induced intestinal symptoms would add to the existing, scant knowledge. Randomized controlled trials focusing on strategies to minimize pelvic radiation-induced symptoms for gynaecological cancer patients are needed. Treatment plans should include consideration of managing working life after treatment.

### Policy implications

The findings highlight the importance of investment in acquiring better technology to deliver smarter and kinder radiotherapy treatment [31, 32]. Radiotherapy treatment centres should provide more personal for radiation therapy treatment planning. More precise and patient-specific optimal radiation doses should be delivered by using magnetic resonance imaging (MRI)-guided radiotherapy. Another opportunity could be combining computerized tomography (CT) scans with ultrasound imaging to create more comprehensive images allowing clinicians to better plan and deliver radiotherapy. The risk of radiation-induced syndromes and work-related outcome parameters should be considered in the planning of pelvic radiotherapy. Future studies should investigate novel interventions and/or rehabilitation programmes to reduce the burden of these syndromes.

**What is already known on this topic?**

- Previous studies and reviews have reported the effect of cancer treatment on both physical and psychological wellbeing and work-related outcomes
- Most studies have focused on breast cancer, prostate cancer, and colorectal cancer survivors and often concentrate on fatigue, pain, anxiety, depression, cognition, physical changes as hair loss, etc.

**What this study adds?**

- This study reports the effect of pelvic radiation-induced gastrointestinal syndromes on the granting of disability pensions among gynaecolcogical cancer survivors
- The study suggests that radiation-induced survivorship syndromes increase the risk of being granted a disability pension
- It also suggests a higher risk of disability pension in cancer survivors several syndromes compared with cancer survivors with fewer syndromes or without syndromes
- Evidence from our study is generally consistent with previous evidence reporting an increased risk of not returning to work among cancer survivors treated with radiotherapy and/or reporting adverse effects of cancer treatment

## Supplementary information

Figure S1. Flow chart of recruitment and selection of gynaecological cancer survivors for this study (PDF 38 kb)

ESM 1 (PDF 171 kb)

ESM 2 (PDF 110 kb)

ESM 3 (PDF 118 kb)

Figure S2. Number (percentage) and risk difference (RD) (95% confidence interval) of disability pension (data taken from the official register) at the 2-year follow-up. Risk differences (CIs) obtained from log-binomial regression analyses using *syndromes* as a predictor. Self-reported symptoms were used to classify survivors having a syndrome. A risk difference of > 0 indicates harm (No. = number). (PNG 1048 kb)

High Resolution Image (TIFF 7.04 mb)

ESM 5 (DOCX 31 kb)

ESM 6 (PDF 78 kb)

## Declarations

### Transparency

The lead author (Adnan Noor Baloch) affirms that this manuscript is an honest, accurate, and transparent account of the study being reported; that no important aspects of the study have been omitted; and that any discrepancies from the study as planned have been explained.
