# Supplementary material for: Disability pension among gynaecological cancer survivors with or without radiation-induced survivorship syndromes
Source: J Cancer Surviv. 2021 Aug 19;16(4):834–43. doi: 10.1007/s11764-021-01077-9 (PMC9300541; doi:10.1007/s11764-021-01077-9)
Supplement: Supplementary file 1 — Flow chart of recruitment and selection of gynaecological cancer survivors for this study (PDF 38 kb) [file 11764_2021_1077_MOESM1_ESM.pdf]

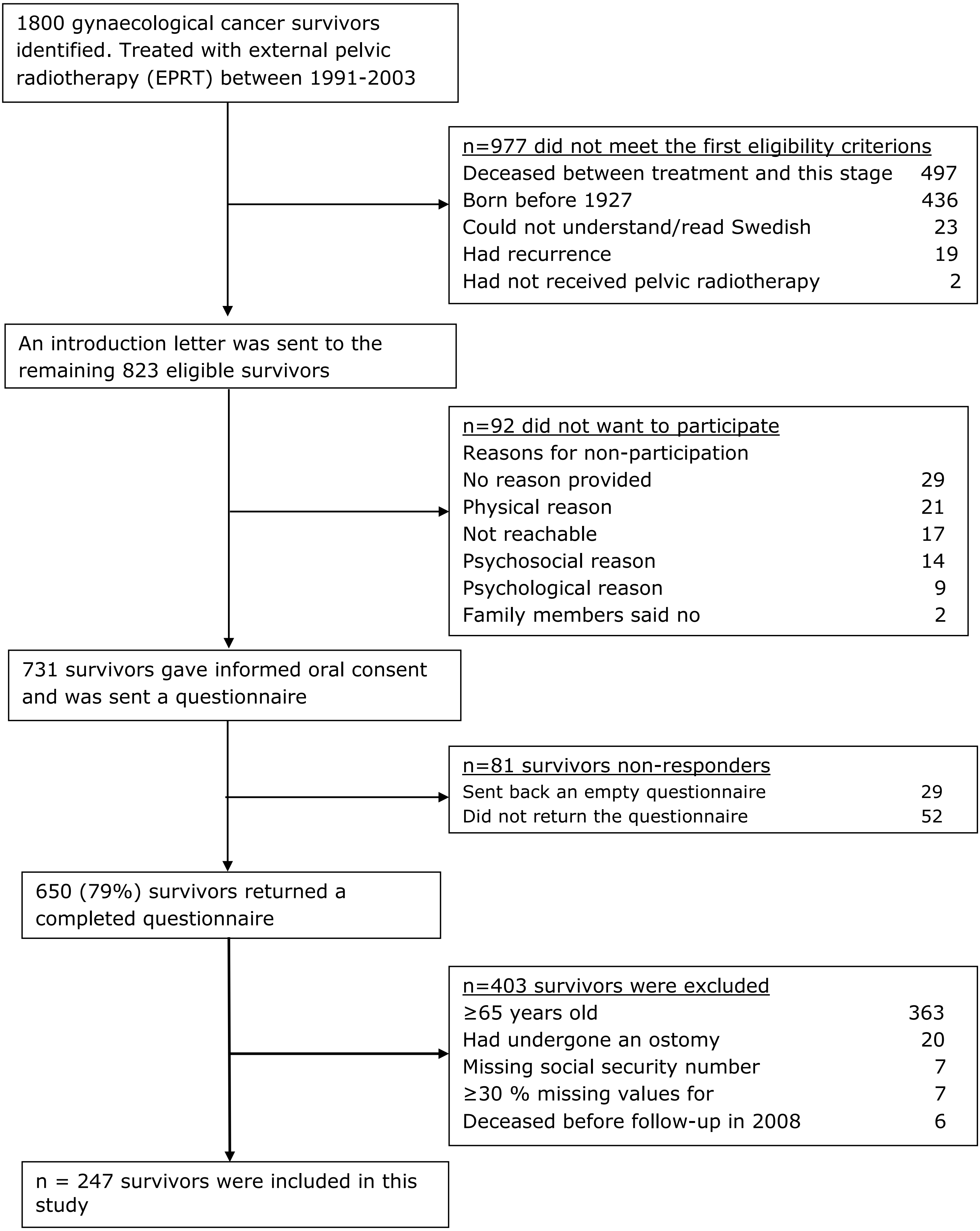
1800 gynaecological cancer survivors identified. Treated with external pelvic radiotherapy (EPRT) between 1991-2003
n=977 did not meet the first eligibility criterions
Deceased between treatment and this stage 497
Born before 1927 436
Could not understand/read Swedish 23
Had recurrence 19
Had not received pelvic radiotherapy 2
An introduction letter was sent to the remaining 823 eligible survivors
n=92 did not want to participate
Reasons for non-participation
No reason provided 29
Physical reason 21
Not reachable 17
Psychosocial reason 14
Psychological reason 9
Family members said no 2
731 survivors gave informed oral consent and was sent a questionnaire
n=81 survivors non-responders
Sent back an empty questionnaire 29
Did not return the questionnaire 52
650 (79%) survivors returned a completed questionnaire
n=403 survivors were excluded
≥65 years old 363
Had undergone an ostomy 20
Missing social security number 7
≥30 % missing values for 7
Deceased before follow-up in 2008 6
n = 247 survivors were included in this study
