# Supplementary material for: Disability pension among gynaecological cancer survivors with or without radiation-induced survivorship syndromes
Source: J Cancer Surviv. 2021 Aug 19;16(4):834–43. doi: 10.1007/s11764-021-01077-9 (PMC9300541; doi:10.1007/s11764-021-01077-9)
Supplement: Supplementary file 2 — (PDF 171 kb) [file 11764_2021_1077_MOESM2_ESM.pdf]

Table S1: Baseline Clinical and demographic data for all gynaecological cancer survivors and for gynaecological cancer survivors with and without syndromes

	All survivors	Gynaecological survivors with				Urgency Syndrome		Leakage Syndrome		Blood Discharge Syndrome	
		no syndrome	one syndrome	two syndromes	three syndromes	Yes	No	Yes	No	Yes	No
	n=247	n=131 (53 %) [a]	n=50 (20%) [a]	n=49 (20%) [a]	n=17 (7 %) [a]	n=91 (37 %) [a]	n=156 (63 %) [a]	n=77 (31 %) [a]	n=170 (69 %) [a]	n=31 (13 %) [a]	n=216 (87 %) [a]
	No. (%)[b]	No. (%)[b]	No. (%)[b]	No. (%)[b]	No. (%)[b]	No. (%)[b]	No. (%)[b]	No. (%)[b]	No. (%)[b]	No. (%)[b]	No. (%)[b]
**Marital Status**											
Married or living with partner	160 (65 %)	86 (66 %)	38 (76 %)	26 (53 %)	10 (59 %)	58 (64 %)	102 (66 %)	46 (60 %)	114 (67 %)	16 (52 %)	144 (67 %)
Widow	12 (5 %)	6 (5 %)	0 (0 %)	4 (8 %)	2 (12 %)	5 (5 %)	7 (5 %)	6 (8 %)	6 (4 %)	3 (10 %)	9 (9 %)
Has partner but lives alone	21 (9 %)	6 (5 %)	6 (12 %)	8 (16 %)	1 (6 %)	11 (12 %)	10 (6 %)	12 (16 %)	9 (5 %)	2 (6 %)	19 (9 %)
Single	53 (22 %)	32 (25 %)	6 (12 %)	11 (22 %)	4 (24 %)	17 (19 %)	36 (23 %)	13 (17 %)	40 (24 %)	12 (32 %)	43 (20 %)
Not stated	1	1					1		1		1
**Education**											
Elementary school	55 (22 %)	26 (20 %)	9 (18 %)	14 (29 %)	6 (35 %)	25 (27 %)	30 (19 %)	22 (29 %)	33 (19 %)	8 (26 %)	47 (22 %)
Secondary school	91 (37 %)	50 (38 %)	16 (32 %)	20 (41 %)	5 (29 %)	30 (33 %)	61 (39 %)	28 (36 %)	63 (37 %)	13 (42 %)	78 (36 %)
College or university	101 (41 %)	55 (42 %)	25 (50 %)	15 (31 %)	6 (35 %)	36 (40 %)	65 (42 %)	27 (35 %)	74 (44 %)	10 (32 %)	91 (42 %)
**Country of birth**											
Sweden	203 (83 %)	109 (84 %)	41 (82 %)	39 (80 %)	14 (82 %)	74 (81 %)	129 (83 %)	64 (83 %)	139 (82 %)	23 (74 %)	180 (84 %)
Other country	43 (17 %)	21 (16 %)	9 (18 %)	10 (21 %)	3 (18 %)	17 (19 %)	26 (17 %)	13 (17 %)	30 (18 %)	8 (26 %)	35 (16 %)
Not stated	1	1					1		1		1
**Place of residency**											
Rural district	27 (11 %)	12 (9 %)	6 (12 %)	5 (10 %)	4 (24 %)	13 (14 %)	14 (9 %)	9 (12 %)	18 (11 %)	6 (19 %)	21 (10 %)
Village/Small town	67 (27 %)	36 (27 %)	11 (22 %)	14 (29 %)	6 (35 %)	26 (29 %)	41 (26 %)	22 (29 %)	45 (26 %)	9 (29 %)	58 (27 %)
> 500.000 citizens	153 (62 %)	83 (63 %)	33 (66 %)	30 (61 %)	7 (41 %)	52 (57 %)	101 (65 %)	46 (60 %)	107 (63 %)	16 (52 %)	137 (63 %)
**Smoking**											
Current smoker	69 (29 %)	34 (26 %)	11 (23 %)	17 (36 %)	7 (41 %)	30 (34 %)	39 (25 %)	24 (33 %)	45 (27 %)	12 (39 %)	57 (27 %)
Former smoker	81 (34 %)	50 (38 %)	11 (23 %)	15 (32 %)	5 (29 %)	25 (28 %)	56 (37 %)	21 (29 %)	60 (36 %)	10 (32 %)	71 (34 %)
Never smoker	92 (38 %)	46 (35 %)	26 (54 %)	15 (32 %)	5 (29 %)	34 (38 %)	58 (38 %)	28 (38 %)	64 (38 %)	9 (29 %)	83 (39 %)
Not stated	5	1	2	2		2	3	4	1		5
**Body Mass Index**											
<=18.5	5 (2 %)	4 (3 %)	0	0	1 (7 %)	1 (1 %)	4 (3 %)	1 (1 %)	4 (2 %)	1 (3 %)	4 (2 %)
18.5–25	123 (52 %)	62 (50 %)	28 (58 %)	26 (53 %)	7 (47 %)	46 (52 %)	77 (52 %)	42 (58 %)	81 (50 %)	13 (45 %)	110 (53 %)
25–30	68 (29 %)	38 (31 %)	12 (25 %)	13 (27 %)	5 (33 %)	24 (27 %)	44 (30 %)	16 (22 %)	52 (32 %)	13 (45 %)	55 (27 %)
>=30	40 (17 %)	20 (16 %)	8 (17 %)	10 (21 %)	2 (13 %)	18 (20 %)	22 (15 %)	14 (19 %)	26 (16 %)	2 (7 %)	38 (18 %)
Not stated	11	7	2		2	2	9	4	7	2	9
**Exercise**											
Never	25 (10 %)	12 (9 %)	4 (9 %)	7 (15 %)	2 (12 %)	12 (13 %)	13 (9 %)	9 (12 %)	16 (10 %)	3 (10 %)	21 (11 %)
At least once a month	38 (16 %)	18 (14 %)	6 (13 %)	12 (25 %)	2 (12 %)	14 (16 %)	24 (16 %)	17 (23 %)	21 (13 %)	5 (17 %)	33 (16 %)
At least once a week	176 (74 %)	97 (76 %)	37 (79 %)	29 (60 %)	13 (76 %)	63 (71 %)	113 (75 %)	49 (65 %)	127 (71 %)	22 (73 %)	154 (74 %)
Not stated	8	4	3	1		2	6	2	6	1	7

**Intercurrent diseases**[c]											
Diabetes mellitus	16 (7 %)	10 (8 %)	3 (6 %)	2 (4 %)	1 (6 %)	6 (7 %)	10 (6 %)	2 (3 %)	14 (8 %)	2 (6 %)	14 (7 %)
Hypertension	56 (23 %)	29 (22 %)	10 (22 %)	11 (22 %)	6 (35 %)	22 (24 %)	34 (22 %)	20 (27 %)	36 (21%)	8 (26 %)	48 (23 %)
Heart failure	3 (1 %)	1 (1 %)	1 (2 %)	1 (2 %)	0	1 (1 %)	2 (1 %)	1 (1 %)	2 (1 %)	1 (3 %)	2 (1 %)
Angina pectoris	5 (2 %)	3 (2 %)	1 (2 %)	0	1 (6 %)	2 (2 %)	3 (2 %)	1 (1 %)	4 (2 %)	1 (3 %)	4 (2 %)
Cardiac infarction	2 (1 %)	1 (1 %)	1 (2 %)	0	0	1 (1 %)	1 (1 %)	0	2 (1 %)	0	2 (1 %)
Ulcerative colitis	2 (1 %)	1 (1 %)	0	1 (2 %)	0	1 (1 %)	1 (1 %)	0	2 (1 %)	1 (3 %)	1 (<1 %)
IBS[d] treatment	12 (5 %)	4 (3 %)	1 (2 %)	4 (8 %)	3 (18 %)	8 (9 %)	4 (3 %)	5 (7 %)	7 (4 %)	5 (16 %)	7 (3 %)
Haemorrhoids treatment	19 (8 %)	8 (6 %)	5 (10 %)	5 (10 %)	1 (6 %)	8 (9 %)	11 (7 %)	7 (9 %)	12 (7 %)	3 (10 %)	16 (8 %)
Lactose intolerance	17 (7 %)	4 (3 %)	6 (12 %)	5 (10 %)	2 (12 %)	12 (13 %)	5 (3 %)	8 (11 %)	9 (5 %)	2 (6 %)	15 (7 %)
Gluten intolerance	5 (2 %)	1 (1 %)	2 (4 %)	1 (2 %)	1 (6 %)	2 (2 %)	3 (2 %)	4 (5 %)	1 (1 %)	1 (3 %)	4 (2 %)
Pelvic organ prolapse	1 (<1 %)	0	0	1 (2 %)	0	1 (1 %)	0	1 (1 %)	0	0	1 (<1 %)
Rheumatism	8 (3 %)	2 (2 %)	2 (4 %)	2 (4 %)	2 (12 %)	5 (6 %)	3 (2 %)	5 (7 %)	3 (2 %)	2 (6 %)	6 (3 %)
Kidney disease	7 (3 %)	3 (2 %)	1 (2 %)	2 (4 %)	3 (18 %)	6 (7 %)	3 (2 %)	4 (5 %)	5 (3 %)	4 (13 %)	5 (2 %)
Lung disease	12 (4 %)	4 (3 %)	1 (2 %)	2 (4 %)	0	3 (3 %)	4 (3 %)	2 (3 %)	5 (3 %)	0	7 (3 %)
Thrombosis	15 (6 %)	6 (5 %)	4 (9 %)	4 (8 %)	1 (6 %)	8 (9 %)	7 (5 %)	5 (7 %)	10 (6 %)	2 (6 %)	13 (6 %)
Osteoporosis	9 (4 %)	3 (2 %)	3 (7 %)	2 (4 %)	1 (6 %)	4 (4 %)	5 (3 %)	5 (7 %)	4 (2 %)	1 (3 %)	8 (4 %)
Psychological disorders	36 (15 %)	11 (8 %)	8 (17 %)	11 (22 %)	6 (35 %)	23 (26 %)	13 (9 %)	17 (23 %)	19 (11 %)	8 (26 %)	28 (13 %)
Neurological disorders	2 (1 %)	0 (0%)	1 (2 %)	1 (2 %)	0	1 (1 %)	1 (1 %)	1 (1 %)	1 (1 %)	1 (3 %)	1 (<1 %)
Joint disorder	59 (24 %)	21 (16 %)	11 (24 %)	20 (41 %)	7 (41 %)	32 (36 %)	27 (18 %)	29 (39 %)	30 (18 %)	11 (35 %)	48 (23 %)
CHD	59 (24 %)	30 (23 %)	11 (24 %)	11 (22 %)	7 (41 %)	24 (27 %)	35 (23 %)	21 (28 %)	38 (23 %)	9 (29 %)	50 (24 %)
**Pelvic floor injury**[ce]											
Yes	38 (16 %)	17 (13 %)	7 (15 %)	10 (20 %)	4 (24 %)	17 (19 %)	21 (14 %)	14 (18 %)	24 (15 %)	8 (26 %)	30 (14 %)
Not stated	6	4	2			1	5	1	5		6
**Delivery**[c]											
Fast (< 5h)	92 (38 %)	48 (38 %)	21 (43 %)	17 (35 %)	6 (35 %)	31 (34 %)	61 (40 %)	31 (40 %)	61 (37 %)	11 (35 %)	81 (38 %)
Slow (> 24 h)	47 (19 %)	19 (15 %)	10 (20 %)	14 (29 %)	4 (24 %)	22 (24 %)	25 (16 %)	21 (27 %)	26 (16 %)	7 (23 %)	40 (19 %)
Vacuum	12 (5 %)	3 (2 %)	4 (8 %)	3 (6 %)	2 (12 %)	8 (9 %)	4 (3 %)	4 (5 %)	8 (5 %)	4 (13 %)	8 (4 %)
Forceps	6 (2 %)	1 (1 %)	2 (4 %)	2 (4 %)	1 (6 %)	3 (3%)	3 (2 %)	2 (3 %)	4 (2 %)	4 (13 %)	2 (1 %)
Episiotomy	60 (25 %)	27 (21 %)	12 (24 %)	15 (31 %)	6 (35 %)	26 (29 %)	34 (22 %)	23 (30 %)	37 (22 %)	11 (35 %)	49 (23 %)
Caesarean	16 (7 %)	8 (6 %)	3 (6 %)	2 (4 %)	3 (18 %)	7 (8 %)	9 (6 %)	5 (6 %)	11 (7 %)	4 (13 %)	12 (6 %)
Breech birth	8 (3 %)	4 (3 %)	4 (8 %)	0	0	1 (1 %)	7 (5 %)	2 (3 %)	6 (4 %)	1 (3 %)	7 (3 %)
Not stated[f]	4	3	1			1	3		4		4
**Child weight at delivery**											
> 4 kg, 1 child	34 (14 %)	21 (16 %)	7 (14 %)	4 (8 %)	2 (12 %)	9 (10 %)	25 (16 %)	9 (12 %)	25 (15 %)	3 (10 %)	31 (14 %)
> 4 kg, 2 children	8 (5 %)	4 (3 %)	1 (2 %)	3 (6 %)	0	3 (3 %)	5 (3 %)	3 (4 %)	5 (3 %)	1 (3 %)	7 (3 %)
**Using any kind of medication**											
Yes	154 (62 %)	68 (52 %)	34 (68 %)	36 (73 %)	16 (94%)	72 (79 %)	82 (53 %)	56 (73 %)	98 (58 %)	26 (84 %)	128 (59 %)
**Taking estrogen**											
Yes	120 (49 %)	70 (53 %)	21 (42 %)	23 (47 %)	6 (35 %)	41 (45 %)	79 (51 %)	27 (35 %)	93 (55 %)	17 (55 %)	103 (48 %)

[a] Number (percentage) of survivors within each category of syndromes [b] Number (percentage) of survivors within each variable [c]Only dichotomous variables in this category. The numbers of negative values are left out. IBS[d] denotes Irritable Bowel Syndrome. [e]Injury inflicted during delivery or at other occasion. [f] Number of survivors for which no information was recorded regarding the delivery variables.
