# Supplementary material for: Disability pension among gynaecological cancer survivors with or without radiation-induced survivorship syndromes
Source: J Cancer Surviv. 2021 Aug 19;16(4):834–43. doi: 10.1007/s11764-021-01077-9 (PMC9300541; doi:10.1007/s11764-021-01077-9)
Supplement: Supplementary file 3 — (PDF 110 kb) [file 11764_2021_1077_MOESM3_ESM.pdf]

Table S2: Frequency count (percentage) of disability pension obtained from the national register. For all cancer survivors (n=247) and after excluding survivors (n=243) dying within 2-years of follow-up

	**Disability pension (yes)**	
	**n = 247[a]**	**n = 243[b]**
	**No. (%)[c]**	**No. (%)[c]**
**Among all survivors**	66 (27 %)	65 (27 %)
**Syndrome[d]**		
**Sum of syndromes[f]**		
Three	9 (53 %)	9 (53 %)
Two	22 (45 %)	22 (45 %)
One	11 (22 %)	11 (22 %)
None	24 (18 %)	23 (18 %)
**Urgency syndrome**		
Yes	35 (38 %)	35 (39 %)
No	31 (20 %)	30 (20 %)
**Leakage syndrome**		
Yes	32 (42 %)	32 (42 %)
No	34 (20 %)	33 (20 %)
**Blood discharge syndrome**		
Yes	15 (48 %)	15 (48 %)
No	51 (24 %)	50 (24 %)
**Excessive gas discharge**		
Yes	20 (36 %)	20 (36 %)
No	46 (24 %)	45 (24 %)
**Excessive mucus discharge**		
Yes	15 (27 %)	15 (27 %)
No	51 (27 %)	50 (27 %)

[a] Including survivors alive at follow-up in 2008 [b] Data obtained after excluding survivors who died within 2-years of follow-up (between 2008 and 2010) [c] Number (percentage) of survivors with disability pension. [d]Self-reported symptoms were used to classify survivors having a syndrome [f] Survivors classified as having several syndromes or one or none.
