# Supplementary material for: Disability pension among gynaecological cancer survivors with or without radiation-induced survivorship syndromes
Source: J Cancer Surviv. 2021 Aug 19;16(4):834–43. doi: 10.1007/s11764-021-01077-9 (PMC9300541; doi:10.1007/s11764-021-01077-9)
Supplement: Supplementary file 4 — (PDF 118 kb) [file 11764_2021_1077_MOESM4_ESM.pdf]

Table S3: Age adjusted Relative risks (RRs) and risk differences (RDs) (95% confidence intervals (CIs)) for disability pension (from national register on disability pension) among survivors with one or more syndromes

Disability pension at 2-year follow-up				
	**Relative risks[a] (95 % CI)**		**Risk differences[b] (95% CI)**	
**Syndrome[c]**	**n = 247[d]**	**n = 243[e]**	**n = 247[d]**	**n = 243[e]**
**Sum of syndromes[f]**				
Three syndrome vs None	**2.7 (1.6 to 4.7)**	**2.8 (1.6 to 4.8)**	**30% (4% to 55%)**	**30% (5% to 56%)**
Two syndrome vs None	**2.5 (1.6 to 4.0)**	**2.6 (1.6 to 4.1)**	**23% (7% to 39%)**	**24% (8% to 40%)**
One syndrome vs None	1.3 (0.7 to 2.4)	1.3 (0.7 to 2.4)	5% (-9% to 18%)	5% (-9% to 19%)
**Urgency vs No urgency syndrome**	**2.0 (1.3 to 3.0)**	**2.0 (1.4 to 3.0)**	**17% (4% to 29%)**	**17% (5% to 30%)**
**Leakage vs No leakage syndrome**	**2.0 (1.4 to 3.0)**	**2.1 (1.4 to 3.0)**	**18% (5% to 31%)**	**18% (5% to 31%)**
**Blood vs No blood discharge syndrome**	**2.1 (1.4 to 3.1)**	**2.1 (1.4 to 3.1)**	**23% (4% to 41%)**	**23% (4% to 42%)**
**Excessive gas discharge vs No excessive gas discharge**	**1.7 (1.2 to 2.6)**	**1.8 (1.2 to 2.7)**	11 % (-4 to 26%)	11 % (-4 to 26%)
**Excessive mucus discharge vs No excessive mucus discharge**	1.1 (0.7 to 1.7)	1.1 (0.7 to 1.7)	1 % (-12% to 14%)	1 % (-12% to 14%)

[a b] Age-adjusted RR and RD (95% CI) obtained from log-binomial regression analyses using *Syndrome* and *Age (in years)* as independent variables, '*None/No*' level of *Syndrome* was used as a reference. [c] Self-reported symptoms were used to build *Syndromes*. [d] Survivors alive at follow-up in 2008. [e] Excluding survivors who died within the 2-years of follow-up (between 2008 and 2010) [f] Survivors classified as having several syndromes or one or none. Bold **numbers** indicate a statistically significant association at 5% level of significance.
