# Supplementary figures and images for: Disability pension among gynaecological cancer survivors with or without radiation-induced survivorship syndromes
Source: J Cancer Surviv. 2021 Aug 19;16(4):834–43. doi: 10.1007/s11764-021-01077-9 (PMC9300541; doi:10.1007/s11764-021-01077-9)

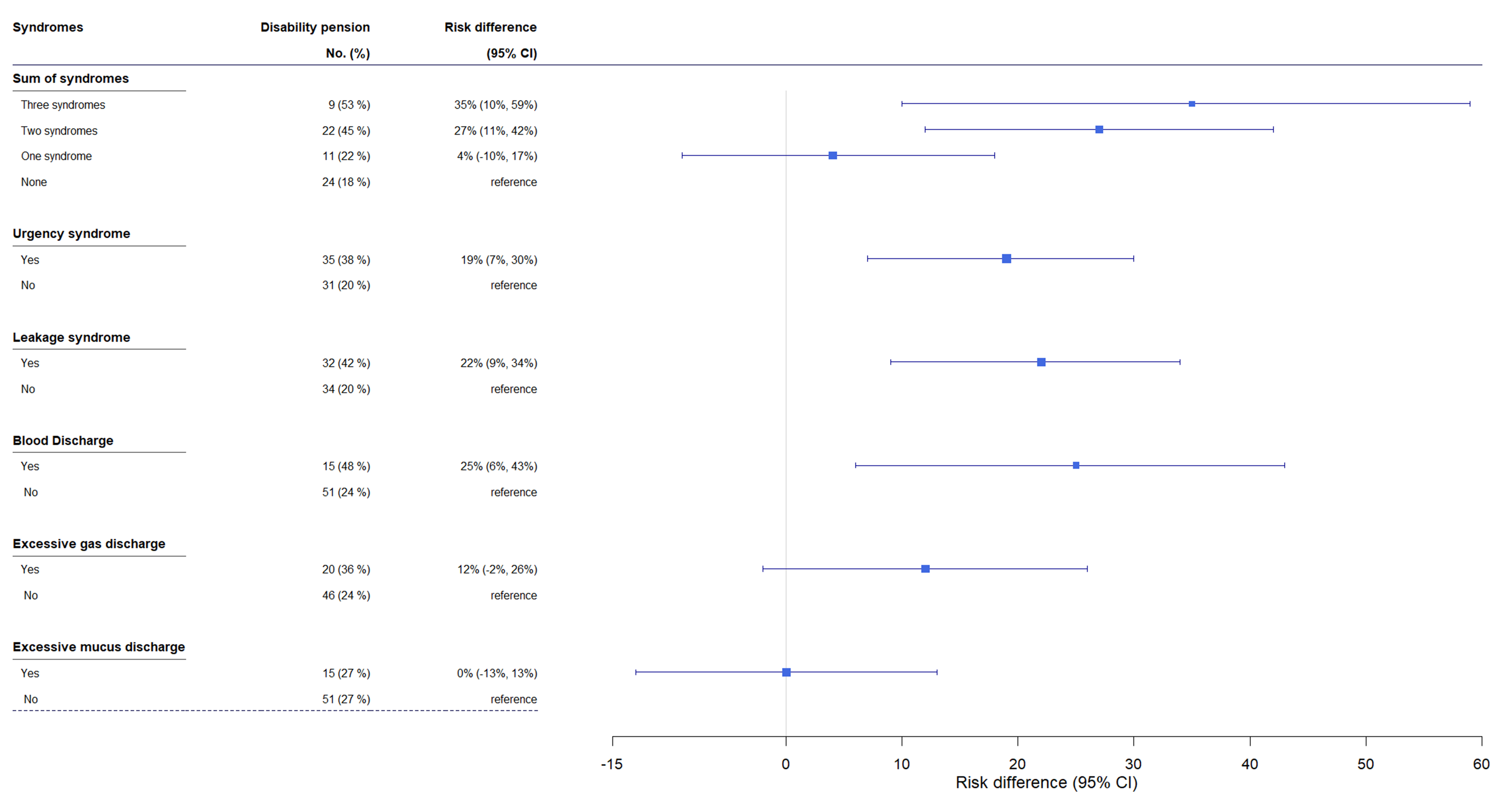

Supplement: Supplementary file 5 — Number (percentage) and risk difference (RD) (95% confidence interval) of disability pension (data taken from the official register) at the 2-year follow-up. Risk differences (CIs) obtained from log-binomial regression analyses using syndromes as a predictor. Self-reported symptoms were used to classify survivors having a syndrome. A risk difference of > 0 indicates harm (No. = number). (PNG 1048 kb) [file 11764_2021_1077_Fig2_ESM.png]

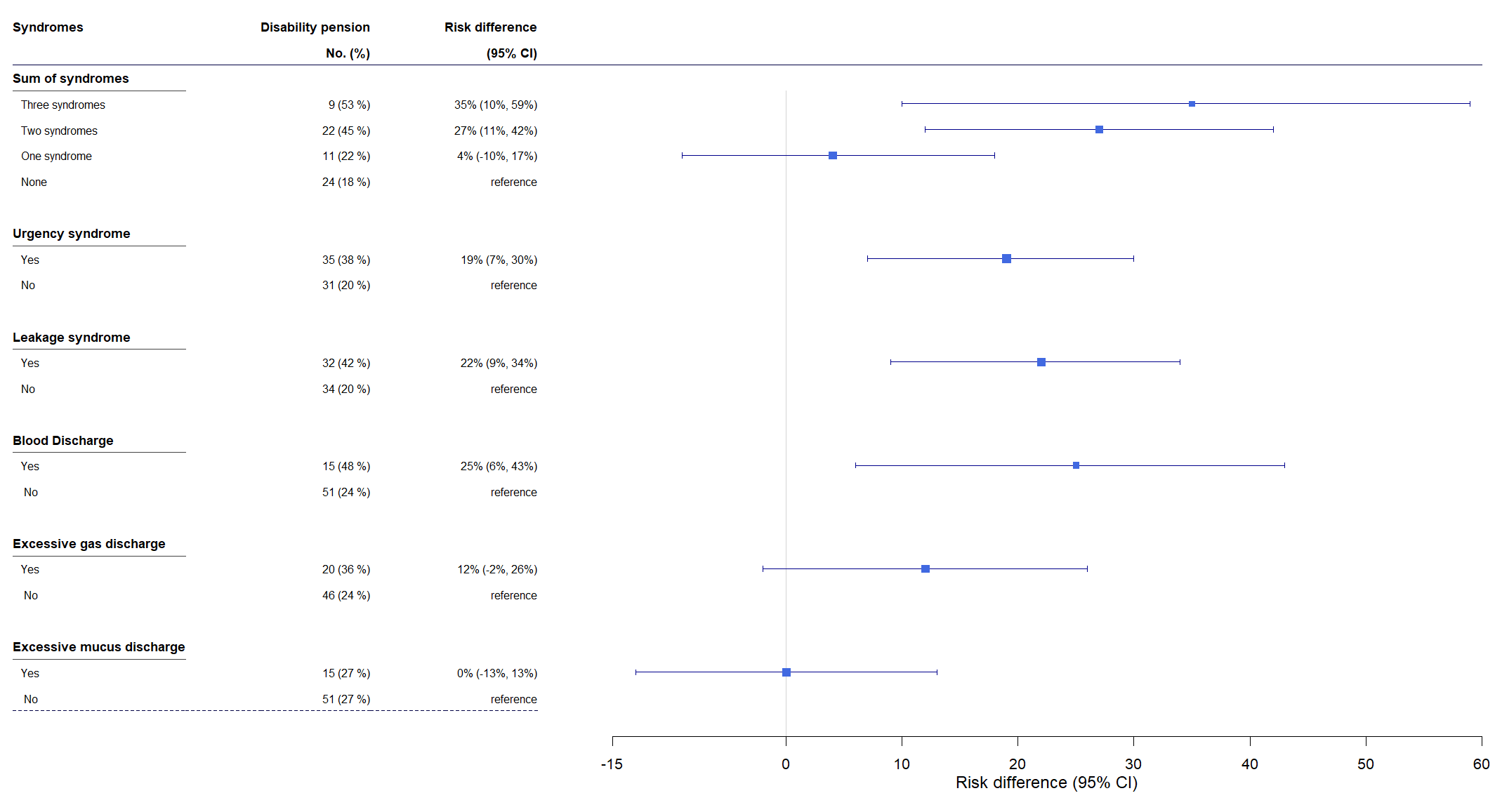

Supplement: Supplementary file 6 — High Resolution Image (TIFF 7.04 mb) [file 11764_2021_1077_MOESM5_ESM.tiff]
