# Supplementary material for: Disability pension among gynaecological cancer survivors with or without radiation-induced survivorship syndromes
Source: J Cancer Surviv. 2021 Aug 19;16(4):834–43. doi: 10.1007/s11764-021-01077-9 (PMC9300541; doi:10.1007/s11764-021-01077-9)
Supplement: Supplementary file 8 — (PDF 78 kb) [file 11764_2021_1077_MOESM7_ESM.pdf]

ClinicalTrials.gov Protocol Registration and Results System (PRS) Receipt
Release Date: May 23, 2019

ClinicalTrials.gov ID: NCT03961217

Study Identification

Unique Protocol ID: ANB_691-17

Brief Title: Return to Work Among Cancer Survivors With Treatment-induced Survivorship Syndromes

Official Title: Return to Work Among Cancer Survivors With Treatment-induced Survivorship Syndromes

Secondary IDs:

Study Status

Record Verification: May 2019

Overall Status: Completed

Study Start: January 1991 [Actual]

Primary Completion: October 2006 [Actual]

Study Completion: December 2016 [Actual]

Sponsor/Collaborators

Sponsor: Sahlgrenska University Hospital, Sweden

Responsible Party: Principal Investigator
Investigator: Gunnar Steineck [gsteineck]
Official Title: Professor / Senior consultant
Affiliation: Sahlgrenska University Hospital, Sweden

Collaborators: Vastra Gotaland Region

Oversight

U.S. FDA-regulated Drug: No

U.S. FDA-regulated Device: No

U.S. FDA IND/IDE: No

Human Subjects Review: Board Status: Approved
Approval Number: 691-17
Board Name: Regional Ethical Review Board, Universities of Gothenburg
Board Affiliation: Universities of Gothenburg
Phone: +46 31 786 6821
Email: anna.fredriksson@epn.gu.se
Address:

Regionala Etikprövningsnämnden i Göteborg
Box 401
405 30 Gothenburg
Sweden

Data Monitoring: No

FDA Regulated Intervention: No

Study Description

Brief Summary: The investigators plan to investigate the consequences of late effects (radiation-induced survivorship syndromes) after radiotherapy in Gynecological and Prostate cancer survivors on return to work (Yes/No) and if RTW happened then time to RTW.

In addition, whether general health, type of work (occupation), work environment factors, individual factors (lifestyle, socioeconomic status etc.), contribute to the adverse late effects of radiotherapy and these Gynecological cancer survivors have a higher risk for disability pension/long term sickness absence (NOT Return to work).

Detailed Description: Occurrence of cancer diagnoses are rising, and both disease and treatments are aggressive. Due to advancement in medical technology, improved therapy and/or early detection the overall survival rates are also improving.

Some of the most common cancer types, such as breast cancer, prostate cancer, cervical cancer, and colorectal cancer have high cure rates when detected early and treated according to best practices. Many of these cancer survivors are of working age and are likely to return to work. Women who survive cervical cancer and men who survive testicular cancer typically have three to four decades left in working life.

However, return to work (RTW) among cancer survivors may not be similar to RTW among long-term sickness absentees due to other diagnoses. Cancer is a life threatening disease and cancer diagnose is a life changing event. The emotional shock after the cancer diagnosis may be associated with low psychological well-being even two years after prostate cancer surgery.

The successful cancer treatment concludes with the lifelong consequences of surgery, irradiation, cytotoxic chemotherapy, biological anticancer substances or other drugs in the treatment. The ionizing radiation that eliminates malignant cells may trigger long-lasting pathophysiological processes in the normal tissue and affect the health of the survivors with lifelong treatment-induced survivorship diseases. In a recent study, Steineck et al, identified five radiation-induced survivorship syndromes affecting bowel health in a cohort of gynecological cancer; urgency syndrome (30%), leakage syndrome (26%), excessive gas discharge(15%), excessive mucus discharge (16%) and blood discharge (10%).

There is a lack of knowledge about how these side effects of cancer treatment affect the degree of work ability and return to work. Clinical experience suggests that many cancer survivors have reduced work ability. There is a need for scientific studies that shed light on the side effects of cancer treatment and their relation to work ability.

Conditions

Conditions: Gynecologic Cancer
Prostate Cancer
Radiation Toxicity

Malignancy
Radiotherapy Side Effect
Radiation Injuries
Cancer Survivors
Radiation Syndrome

Keywords: Return to work
Disability pension
Sickness Absence
Employability
Fitness to work
Wellbeing
Radiotherapy Side Effect
Cancer Survivors

Study Design

Study Type: Observational [Patient Registry]

Observational Study Model: Case-Only

Time Perspective: Prospective

Biospecimen Retention: None Retained

Biospecimen Description:

Enrollment: 2135 [Actual]

Number of Groups/Cohorts: 3

Target Follow-Up Duration: 5 Years

Groups and Interventions

Groups/Cohorts	Interventions
Gynecological Cases<br>Gynecological Cancer survivors with treatment induced survivorship syndroms treated pelvic radiotherapy at<br>1. Radiumhemmat, Karolinska University Hospital and<br>2. Jubileumskliniken at Sahlgrenska University Hospital in Sweden.	Radiation: Radiotherapy<br>Radiotherapy as part of cancer treatment
Prostate Cases<br>Prostate Cancer survivors treated with radiotherapy for localized prostate cancer at Sahlgrenska University Hospital, Gothenburg, Sweden	Radiation: Radiotherapy<br>Radiotherapy as part of cancer treatment
Gynecological Rehab Cases<br>Gynecological Cancer survivors with treatment induced survivorship syndroms treated pelvic radiotherapy	Radiation: Radiotherapy<br>Radiotherapy as part of cancer treatment

Outcome Measures

Primary Outcome Measure:

1. Return to work (RTW)
   Information on Sickness absence for more than 14 days was obtained from the official registries in Sweden.

   [Time Frame: Five - Ten years after mapping Radiation-induced survivorship syndromes]

Secondary Outcome Measure:

2. Time to Return to work
   Sickness absence (more than 14 days) until the survivor returned to work. Also obtained from the official registries in Sweden.

[Time Frame: Five - Ten years after mapping Radiation-induced survivorship syndromes]

Eligibility

Study Population: Cohort of women treated with external pelvic radiotherapy for a gynaecological malignancy and men treated with radiotherapy for prostate cancer at two large oncology clinics in Sweden.

Sampling Method: Non-Probability Sample

Minimum Age: 25 Years

Maximum Age: 60 Years

Sex: All

Gender Based: Yes
Gyaecological cancer survivors Prostate cancer survivors

Accepts Healthy Volunteers: No

Criteria: Inclusion Criteria:

a. Age 25 to 60 years (working age)
b. Employed at time of diagnosis
c. Treated for cancer
d. Suffering from at least one of Radiation-induced survivorship syndromes
e. First cancer & first time treatment for cancer,

Exclusion Criteria:

a. No other chronic disease
b. Chronic intestinal illness/surgery
c. Stage IV cancer
d. Recurrent cancer/Relapse

Contacts/Locations

Central Contact Person: Gunnar Steineck, MD Ph.D
Telephone: +46 31 3428503
Email: gunnar.steineck@oncology.gu.se

Central Contact Backup: Adnan Noor Baloch, M. Sc
Telephone: +46 31 786 3205
Email: adnan.noor.baloch@gu.se

Study Officials: Gunnar Steineck
Study Principal Investigator
Clinical Cancer Epidemiology, Sahlgrenska Academy, Gothenburg, Sweden

Locations: **Sweden**
Jubileumskliniken, Sahlgrenska University Hospital
Gothenburg, Sweden, 413 45
Contact: Johanna Svensson, MD Johanna.Svensson@vgregion.se

IPDSharing

Plan to Share IPD: No

References

Citations: Steineck G, Skokic V, Sjöberg F, Bull C, Alevronta E, Dunberger G, Bergmark K, Wilderäng U, Oh JH, Deasy JO, Jörnsten R. Identifying radiation-induced survivorship syndromes affecting bowel health in a cohort of gynecological cancer survivors. PLoS One. 2017 Feb 3;12(2):e0171461. doi: 10.1371/journal.pone.0171461. eCollection 2017. PubMed 28158314

Dunberger G, Lind H, Steineck G, Waldenström AC, Nyberg T, Al-Abany M, Nyberg U, Vall-Lundqvist E. Self-reported symptoms of faecal incontinence among long-term gynaecological cancer survivors and population-based controls. Eur J Cancer. 2010 Feb;46(3):606-15. doi: 10.1016/j.ejca.2009.10.023. Epub 2009 Nov 18. PubMed 19926277

Alsadius D, Hedelin M, Johansson KA, Pettersson N, Wilderäng U, Lundstedt D, Steineck G. Tobacco smoking and long-lasting symptoms from the bowel and the anal-sphincter region after radiotherapy for prostate cancer. Radiother Oncol. 2011 Dec;101(3):495-501. doi: 10.1016/j.radonc.2011.06.010. Epub 2011 Jul 5. PubMed 21737169

Lind H, Waldenström AC, Dunberger G, al-Abany M, Alevronta E, Johansson KA, Olsson C, Nyberg T, Wilderäng U, Steineck G, Åvall-Lundqvist E. Late symptoms in long-term gynaecological cancer survivors after radiation therapy: a population-based cohort study. Br J Cancer. 2011 Sep 6;105(6):737-45. doi: 10.1038/bjc.2011.315. Epub 2011 Aug 16. PubMed 21847122

Links: URL: https://doi.org/10.1016/j.ejca.2009.10.023
Description Self-reported symptoms of faecal incontinence among long-term gynaecological cancer survivors and population-based controls

URL: https://dx.doi.org/10.1371%2Fjournal.pone.0171461
Description Identifying radiation-induced survivorship syndromes affecting bowel health in a cohort of gynecological cancer survivors

URL: https://doi.org/10.1016/j.radonc.2011.06.010
Description Tobacco smoking and long-lasting symptoms from the bowel and the anal-sphincter region after radiotherapy for prostate cancer

URL: https://www.nature.com/articles/bjc2011315
Description Late symptoms in long-term gynaecological cancer survivors after radiation therapy: a population-based cohort study

Available IPD/Information:
